# Supplementary material for: Selective Internal Radiotherapy (SIRT) and Chemosaturation Percutaneous Hepatic Perfusion (CS-PHP) for Metastasized Uveal Melanoma: A Retrospective Comparative Study
Source: Cancers (Basel). 2023 Oct 11;15(20):4942. doi: 10.3390/cancers15204942 (PMC10605323; doi:10.3390/cancers15204942)
Supplement: Supplementary file 1 [file cancers-15-04942-s001.zip › cancers-2541924-supplementary.pdf]

Supplementary Materials

Table S1. Overall survival of the study population from the timepoint of study treatment in three different approaches to available data.

OS in Days	Estimation		SIRT	CS-PHP
from treatment	restrictive	mean ± SD	349.74 ± 289.33	365.08 ± 325.91
		median (range)	250 (19–1254)	259 (26–1110)
	moderate	mean ± SD	409.94 ± 406.96	628.71 ± 512.90
		median (range)	300.5 (19–1912)	516 (5–1836)
	optimistic	mean ± SD	581.79 ± 530.73	756.61 ± 499.31
		median (range)	527 (19–2164)	714.5 (26–1830)

Table S2. Progression-free survival from the timepoint of study treatment in three different approaches to available data.

PFS in Days	Estimation		SIRT	CS-PHP
from treatment	restrictive	mean ± SD	349.74 ± 289.32	365.08 ± 325.91
		median (range)	250 (19–1254)	259 (26–1110)
	moderate	mean ± SD	303.88 ± 392.28	527.71 ± 473.43
		median (range)	127.5 (19–1912)	408.5 (3–1809)
	optimistic	mean ± SD	475.74 ± 554.53	706.79 ± 532.11
		median (range)	222.5 (19–2164)	686.5 (3–1830)

Table S3. Overall survival and progression-free survival from the timepoint of study treatment in months in the literature.

Study	SIRT OS	SIRT PFS	CS-PHP OS	CS-PHP PFS
This study	9.90	4.16	16.96	13.43
Abbott A.M. [35]	9.69	1.8		
Alexander H. [20]	12.3			
Meijer T. [25]			19.1	7.6
Dewald C. [24]			18.4	8.4
Hughes M. [23]			10.6	5.4
Modi S. [36]			14.9	8.4
Brüning R. [37]			16.7	
Karydis I. [38]			15.3	8.1
